# Effects of Redox Homeostasis and Mitochondrial Damage on Alzheimer’s Disease

**DOI:** 10.3390/antiox12101816

**Published:** 2023-09-30

**Authors:** Yi-Hsuan Wu, Hsi-Lung Hsieh

**Affiliations:** 1 Research Center for Chinese Herbal Medicine, College of Human Ecology, Chang Gung University of Science and Technology, Taoyuan 333, Taiwan or yhwu03@mail.cgust.edu.tw; 2 Department of Nursing, Division of Basic Medical Sciences, Graduate Institute of Health Industry Technology, Chang Gung University of Science and Technology, Taoyuan 333, Taiwan; 3 Department of Neurology, Chang Gung Memorial Hospital, Taoyuan 333, Taiwan

**Keywords:** Alzheimer’s disease, tau, β-amyloid, APOE4, redox homeostasis, mitochondrial dysfunctions

## Abstract

Bioenergetic mitochondrial dysfunction is a common feature of several diseases, including Alzheimer’s disease (AD), where redox imbalance also plays an important role in terms of disease development. AD is an age-related disease and begins many years before the appearance of neurodegenerative symptoms. Intracellular tau aggregation, extracellular β-amyloid (Aβ) deposition in the brain, and even the APOE4 genotype contribute to the process of AD by impairing redox homeostasis and mitochondrial dysfunction. This review summarizes the evidence for the redox imbalance and mitochondrial dysfunction in AD and demonstrates the current therapeutic strategies related to mitochondrial maintenance.

## 1. Introduction

According to the predictions from the United Nations, globally, by 2030, one in six individuals will be aged 60 years or over, and the population will double by 2050. Such data consistently indicate an increasing percentage of older adults. These phenomena have increased the incidence of age-related neurodegenerative diseases such as dementia [1]. Alzheimer’s disease (AD) is the most common dementia, presenting an increasing prevalence with age over 65 and a doubling incidence every five years [2]. AD is considered a common cause of death in elderly people and is characterized by cognitive impairment with the involvement of language, memory, communication, judgment, and reasoning. However, there is no cure for AD, although there exist treatments available for the improvement of some symptoms. Some lifestyle interventions, such as a healthy diet (calorie restriction and time-restricted eating), could play a potential role in delaying AD progression [3]. Moreover, higher education, leisure activities, e.g., playing musical instruments and reading, the use of anti-inflammatory agents, and regular aerobic exercise, are also known to decrease AD risk. To date, the complex pathophysiology of AD remains uncertain. Amyloid and tau deposits, metabolic dysfunction, and hyper-neuroinflammatory responses, including increased expression of tumor necrosis factor alpha (TNF-α), interleukin-1β (IL-1β), IL-6, IL-8, and IL-10, caused by AD progression are involved in the exacerbation of synaptic loss and neuronal death [4].

AD is a progressive neural disease caused by neuronal cell death, and it begins many years before neurodegenerative symptoms appear. The current studies indicate that AD is primarily caused by the abnormal build-up of proteins, where amyloid-β (Aβ) protein, which is derived from a well-known protein, amyloid precursor protein (APP), and tau protein are deposited around and within brain cells by forming plaques and tangles. In clinical terms, AD is characterized by abundant amyloid plaques, neurofibrillary tangles, granulovacuolar degeneration, and synapse loss [5]. Amyloid plaques are A-peptide spherical microscopic lesions that are deposited in the brain’s grey matter. The function of the tau protein is stabilization of axonal microtubules, which are essential for intracellular transport. Neurofibrillary tangles are fibrillary intracytoplasmic structures formed through tau aggregation. Granulovacuolar degeneration occurs in hippocampal pyramidal cells. Some studies have demonstrated that cognitive decline of AD correlates with the decreased density of pyramidal presynaptic boutons [6,7]. Loss of synapses is highly correlated with cognitive impairment in AD patients, manifesting as the early disease progress that precedes neuronal loss [8]. However, the causes of this process are still unclear, where prevailing evidence supports that the amyloid cascade hypothesis plays an important role in AD pathology. The transmembrane protein APP is followed by consecutive protease processing to release the amyloid-β peptide, including pathogenic species Aβ42. Aβ42 assembles into oligomers (2–100 units) and protofibrils (>100 kDa) and deposits as senile plaques, causing a neurotoxic effect [9]. The recent lipid-chaperone hypothesis suggests that the presence of free lipids in the aqueous phase, not bound to membranes, forms a stable complex with the Aβ protein, which is then transported into the bilayer and may play roles in destroying cell membrane integrity [10]. When the brain cells are affected, acetylcholine, the main neurotransmitter in the brain, is significantly decreased, thus leading to organ dysfunction by disrupting processes vital to neurons and their networks, including metabolism, communication, and repair. Over time, age-based atrophy occurs in different brain areas, including the entorhinal cortex, hippocampus, and cerebral cortex, typically starting in the entorhinal cortex and hippocampus.

Since AD is a kind of age-related disease, aging seems to be the main risk factor for brain disorder processes. Compelling evidence indicates that the intracellular aggregation of tau and extracellular Aβ deposition in the brain are the key features of AD [11]. Interestingly, these proteins also accumulate in the brains of people aged over 50 years without cognitive impairment, indicating that the abnormal accumulation of these proteins might represent preclinical stages of AD through proteinopathy-independent events [12]. The hallmarks of aging, described by Lopez-Otin et al. in 2013, include genomic instability, telomere attrition, epigenetic alterations, the loss of proteostasis, deregulated nutrient sensing, mitochondrial dysfunction, cellular senescence, stem cell exhaustion, and altered intercellular communication [13]. Among those hallmarks, cellular senescence has recently been emphasized as potentially playing a role in the early stages of AD [14]. Cellular senescence is inevitable as a consequence of damage accumulation due to aging [15]. The organism’s metabolic state could be changed due to the accumulation of deleterious damage, thus triggering sustained inflammatory stress activation, such as telomere shortening, the irremediable accumulation of oxidative stress, and the impairment of repair mechanisms. Aging causes brain function to decline over time, and oxidative stress accumulation has been proposed to play an important role in AD progression [16]. Cellular molecular targets affected by oxidative stress include nuclear and mitochondrial DNA, proteins, lipids, mitochondrial dynamics and function, energy homeostasis, etc. Such abnormal cellular metabolism could affect the accumulation of Aβ and phosphorylated Tau proteins, which may further aggravate mitochondrial dysfunction and reactive oxygen species (ROS) production, contributing to a vicious cycle. Moreover, ROS, Aβ, and pTau may independently or synergistically affect the activity of N-methyl-D-aspartate (NMDA) receptors, which function in coordination with α-amino-3-hydroxy-5-methyl-4-isoxa-zolepropionic acid (AMPA) receptors to regulate the excitatory synaptic transmission and plasticity for the functions of learning and memory [17,18]. Although the function of NMDA receptors could decline with age in vivo, the expression of neurotoxic Aβ in AD mice has been shown to reduce the amount of NMDA receptors in neurons and brain tissue [19], which may trigger excitotoxicity, stress-related signaling pathways, increase in oxidative stress, impaired energy metabolism, and the dysregulation of neuronal development and plasticity [20,21].

## 2. Redox Homeostasis in Alzheimer’s Disease

**Figure 1:**
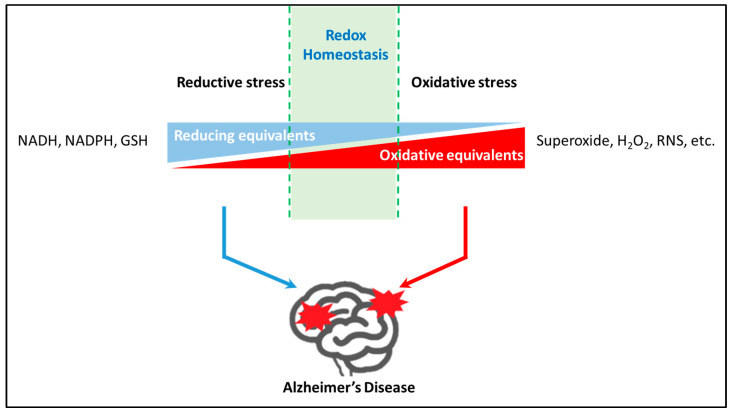
The paradoxical effect of cellular redox status on AD progression. Oxidative and reductive stress are dual dynamic phases due to the imbalance between the production of oxidants (e.g., ROS) and antioxidant defenses with the aberrant increase in reducing equivalents. The human brain is an extremely oxidative organ accounting for 20% of oxygen consumption. Therefore, the regulation of oxidants and antioxidant systems to maintain redox homeostasis is important in the brain. APOE4 genotype, a well-established AD biomarker, makes individuals suffer reductive status for a long time until the onset of AD and develops oxidative stress in their later lives. All these phenomena demonstrate that redox homeostasis plays a vital role in AD development. However, the detailed mechanism needs to be further elucidated.

The mammalian brain is an extremely oxidative organ accounting for a large percentage of oxygen consumption, which is about 20% in humans [22]. In living organisms, oxidants and antioxidant systems play a counteracting role in regulating redox homeostasis within the body. However, redox status may shift towards greater production of pro-oxidants, termed “oxidative stress”, or greater reducing power, termed “reductive stress”, upon the impairment of the endogenous antioxidant system by increasing the levels of reduced NAD^+^ (NADH), reduced NADP^+^ (NADPH), and GSH [23]. There are three major classes of antioxidant enzymes in the body, including catalases, superoxide dismutase (SOD), and glutathione peroxidases (GPx). SOD scavenges superoxide radicals by converting them into hydrogen peroxide (H_2_O_2_), which can be converted into H_2_O by catalase. GPx is involved in the reduction of several peroxides, such as H_2_O_2_, lipid hydroperoxides, and other organic hydroperoxides. Interestingly, evidence has shown that these antioxidant enzymes are dysregulated in AD patients or animal models. In the culture systems of neuroblastoma and hippocampal neurons, Aβ increases the levels of hydrogen peroxide [24,25] through direct binding to catalase and a subsequent decrease in enzyme activities [26]. These in vitro findings demonstrate that catalase—Aβ interaction may play a vital role in the development of oxidative stress in AD. Data from the research on AD animal models and postmortem AD brains have shown impaired SOD1 activity, indicating a decrease in SOD1 that drives AD neuropathology [27]. Moreover, evidence suggests that GPx-1 (Se-dependent glutathione peroxidase-1), the most abundant isoform of GPx in the brain, provides protection against Aβ(1–42)-induced memory impairment through the activation of M1 mAChR-dependent CREB/BDNF signaling [28]. The balance of cellular redox status is shown in Figure 1. The impairment of redox homeostasis may contribute to the progression of AD with paradoxical effects.

Because events leading to AD can take years, or even decades, to manifest, the easily accessible biomarkers of AD are of interest to clinicians [29,30]. Since redox homeostasis is highly controlled in the brain, oxidative or reductive stress may contribute to the development of AD. In humans, the most established biomarker concerning reductive stress increasing the risk of AD is the APOE4 genotype [31], which is common in Africans and Caucasians [32]. Individuals with the APOE4 allele experience reductive status long before the onset of the disease or before the occurrence of mild cognitive impairment [33]. APOE is a key glycoprotein considered to regulate cholesterol transport from one tissue to another [34]. In the brain, APOE is mainly produced by astrocytes and transports cholesterol to neurons. Human APOE exists in one of three major isoforms (E2, E3, and E4), where the APOE4 genotype enhances the deposition of beta-amyloid-forming senile plaques and cerebral amyloid angiopathy (CAA) [35]. Evidence indicates that the APOE4 variant leads to the degeneration of brain capillary pericytes and accelerated breakdown of the blood–brain barrier (BBB) [36]. Interestingly, APP/PS1 transgenic mice, another animal model of AD, are affected by greater reductive stress at a young age [33]. All this evidence indicates that the occurrence of reductive status before or during the progression of AD symptoms is important and functions as an early marker of the disease. 

In addition to reductive stress, oxidative stress plays an even more important role in the development of AD. The ROS in biological systems were first described by Commoner et al. in 1954 [37]. Due to their characteristics of a very short life span and rapid reactions with other molecules, ROS can be associated with the regulation of physiological functions, such as developmental processes and cell singling pathways. ROS generation can be mediated by exogenous and endogenous sources. The major exogenous sources of ROS include environmental pollution, UV radiation, cigarette smoking, certain foods, and toxic chemicals. The endogenous sources of ROS can be generated by different cellular organs, such as peroxisomes, mitochondria, and the endoplasmic reticulum. These active molecules target cellular macromolecules, such as proteins, lipids, and nucleotides, resulting in the impairment of organ functions [38]. The level of 8-iso-prostaglandin F2α (8-iso-PGF2α), a marker of lipid peroxidation, is significantly correlated with neurodegenerative impairment in AD and all-cause dementia [39,40]. 

Being highly sensitive and susceptible to oxidative stress due to their high intake of oxygen and the relative scarcity of antioxidant enzymes, neuronal cells are easily affected by aging as compared with other body tissues [41]. Aging is a natural process of life that maintains the accumulation of somatic mutations with oxidative stress production at any time. The phenotype of aged cells demonstrates futile ROS regulation in the mitochondrial electron transfer chain and changing ROS signaling [42]. Current studies also indicate that the oxidative damage of macromolecules and their products is continuously built up in the brain with the passage of time [43,44]. Furthermore, tau proteins are easily phosphorylated or aggregated in the brain during aging, where the aggregates bind to Fe^3+^, which results in the formation of neurofibrillary tangles [45]. Interestingly, Aβ peptide can also chelate with metal ions, such as Cu^2+^, Zn^2+^, and Fe^3+^, which may produce H_2_O_2_ and toxic OH radicals via metal-mediated catalysis [46]. Lipid peroxidation and oxidative protein damage have been also observed in AD patients, which can induce neuronal death through different mechanisms [47,48]. All these studies indicate that oxidative stress plays a vital role in AD progression.

## 3. The Contribution of Mitochondrial Damage to Neuroinflammatory Response

Mitochondria, the critical intracellular source of energy, are not static organelles and create a complex and highly dynamic network inside cells [49]. This dynamism includes changes in shape and mitochondrial movement along the cytoskeleton. The process of mitochondrial dynamics is tightly regulated by mitochondrial fusion and fission events [50]. The generation of ATP occurs in the inner mitochondrial membrane through oxidative phosphorylation, where free radicals are also produced as a by-product. Regardless of whether there are endogenous or exogenous factors leading to redox imbalance, mitochondria are the main organelles for maintaining cellular homeostasis which serves to modulate many biochemical processes. In addition to the production of energy, mitochondria also play roles in the biosynthesis of fatty acids, calcium buffering, and integration functions in regulating cell signaling, such as cell functions and immune response [51]. 

Since the human brain requires high metabolic energy for its function, neurons are extremely dependent on mitochondrial energy production, countering their limited glycolytic capacity [52]. Due to the particular morphology of neurons, with their extending axons and dendrites, mitochondria are spread throughout the cells, particularly in the synapses, which have the highest energy demand. Given the high energy demand and communication of neurons, suitable mitochondrial dynamics are important to allow for the function and transport of mitochondria. When a neurotransmitter is released by a synapse, the ion channels need to be opened to permit ion influx through the consumption of high energy. Thus, mitochondrial trafficking is vital for neurons’ survival. Moreover, the mitochondrion plays a pivotal role in the maintenance of neuronal functions by regulating apoptotic pathways, intracellular calcium homeostasis, cell cycle regulation, and synaptic plasticity [53]. The mitochondrion is involved in the modulation of polarity by reducing the Ca^2+^ concentration, thereby promoting rapid neuronal growth and differentiation [54]. During the differentiation of axons and dendrites, growth and the synaptic junction can be influenced by mitochondrial dynamics, where mitochondrial fusion joins two mitochondria into one and fission separates one into two. Fusion is coordinated on the outer mitochondrial membrane (OMM) and the inner mitochondrial membrane (IMM) by the mitofusins (MFN1 and MFN2) and optic atrophy 1 (OPA1), respectively. Fission begins when the endoplasmic reticulum (ER) is recruited to the mtDNA-marked site. Next, multiple OMM-bound proteins (FIS1, MFF, MiD49, and MiD51) recruit DRP1 to the surface of the mitochondria, aiding in ER-mediated constriction [55].

**Figure 2:**
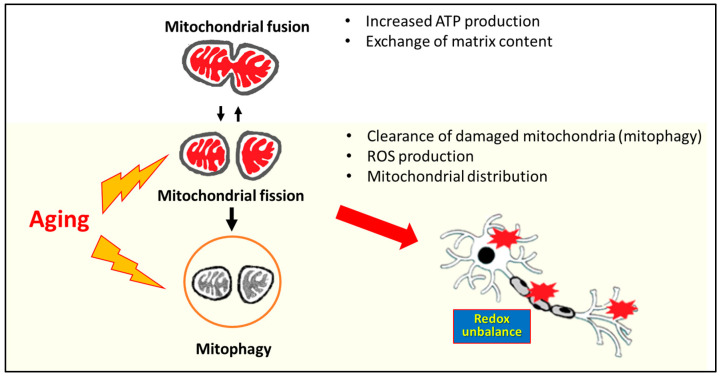
Mitochondrial dynamics (fission, fusion, transport, and mitophagy) are important in cellular functions, such as energy production, cell division, cell differentiation, and cell death. Aging (yellow area) increases mitochondrial fission and impairs mitophagy formation, leading to changes in mitochondrial dynamics and redox unbalance by the increase of oxidative stress and contributing to cell apoptosis. Mitochondria have been thought to contribute to aging through the accumulation of mitochondrial DNA (mtDNA) mutations, which result in respiratory chain deficiencies and increased ROS generation. Increased oxidative stress encourages the oxidation of macromolecules and causes neuronal cell apoptosis.

The degradation of damaged cellular components is called autophagy, where mitophagy is the specialized form of autophagy for mitochondria removal [56]. The proteins involved in modulating mitochondrial dynamics are also responsible for the functions of mitophagy [56]. The function of mitophagy is to remove damaged or dysfunctional mitochondria [57]. However, the impaired mitophagy and altered clearance contribute to the deleterious accumulation of dysfunctional and structurally aberrant mitochondria. Evidence demonstrates that mitophagy induction promotes IL-10 secretion and reduces TNF-α expression in microglia and decreases the inflammatory response in macrophages [58]. Furthermore, mitophagy can also overcome the immune responses during neurodegenerative diseases. However, the mitochondrial dysregulation by impaired mitochondrial dynamics and mitophagy can cause a redox to unbalance (Figure 2), which may encourage the oxidation of macromolecules, such as mtDNA, proteins, and lipids.

Mitochondrial DNA is in proximity to the electron transport chain. Mitochondria are also thought to contribute to aging through the accumulation of mitochondrial DNA (mtDNA) mutations [59], which result in respiratory chain deficiencies and increased ROS generation, all of which lead to the induction of apoptosis [60]. Interestingly, mtDNA also plays the role of activators for different pattern recognition receptors upon release into the cytoplasm or even the extracellular milieu. The landmark study of Collins et al. demonstrated that mtDNA injection into joints induced localized inflammation and arthritis in a mice model [61]. The release of mtDNA due to mitochondria stress or cell death could activate innate immunity via the cGAS-STING, TLR9, and inflammasome formation pathways, leading to cell inflammation, such as NF-κB-mediated proinflammatory response, caspase-1-dependent inflammation, and robust type I interferon responses [61]. Due to this, mtDNA-mutation-induced oxidative stress can contribute to the pathogenesis of chronic diseases, such as cancer, diabetes, and aging. Interestingly, mitochondria-possessed dsRNA is also with potent immunogenicity [62]. Due to mitochondrial transcription, dsRNA is formed by the heavy and light strands of mtDNA [63] and can be detected in the cytoplasm of mitochondria. The accumulation of dsRNA is then degraded by two mitochondrial enzymes, helicase SUV3, and polynucleotide phosphorylase PNPase. However, when these mitochondrial functions are depleted, the dsRNA may activate a type I interferon response through the MDA5 receptor [64]. Furthermore, mitochondrial molecules released into the extracellular space have been shown to initiate proinflammatory responses from non-neuronal glial cells, contributing to chronic neuroinflammation and accelerating the degeneration of neurons [65].

## 4. Mitochondria Dysfunction in Alzheimer’s Disease

Mitochondrial dysfunction causes most neurodegenerative disorders, such as AD, PD, and Huntington’s diseases [66]. However, there exist mitochondrial genetic factors for PD and Huntington’s diseases but less for AD [67,68,69]. Although there is no genetic proof to demonstrate the relationship between mitochondrial dysfunction with AD, the functions of mitochondria are indeed dysregulated in AD patients [70]. Intracellular neurofibrillary tangles and Aβ deposition in the brain are the two major pathological features in AD patients. Previous studies have indicated that mitochondria could be a possible therapeutic target of AD [70]. Intracellular neurofibrillary tangles and Aβ deposition in the brain are the two major pathological features in AD patients. Furthermore, AD is associated with the loss of synapses and neurons. Although there are several molecular and cellular events involved in AD pathogenesis, mitochondrial dysfunction and synaptic damage represent the main early-onset events of AD [71,72], which may be due to the mutant of Aβ precursor protein and aging. Age-mediated Aβ accumulation in synaptic mitochondria could interfere with synaptic functions. Interestingly, mitochondrial trafficking is frequently interrupted in the neurons of AD patients [73]. Moreover, age-dependent oxidative stress contributes to AD development, where mitochondria act as the main source of ROS production [74]. Therefore, several studies have indicated that mitochondria could be a possible therapeutic target of AD [75,76]. 

The dysregulation of mitochondrial dynamics also contributes to AD development through impaired synaptic plasticity and neural cell maintenance [77]. Besides this, defective biogenesis and transport of mitochondria along axons were observed in AD patient-derived neuronal cells and AD mice models, resulting in increased fission activities [78]. The study by Wang et al. found that mitochondria were distributed away from axons in AD pyramidal neurons with altered levels of mitochondrial proteins (reduced expression of Drp1, OPA1, Mfn1, and Mfn2 and increased expression of Fis1) [79]. Several studies have demonstrated that increased fragmentation in neuronal cells could be observed upon DRP1 overexpression [80,81]. Furthermore, Aβ can promote mitochondrial fission by increasing posttranslational modification of Drp1, such as O-GlcNAcylation [82] and phosphorylation by Akt activation [83]. Moreover, MFN2 has been found to be downregulated in the hippocampus and cortex of AD patients [79], and suppression of MFN2 expression by microRNA-195 in SAMP8 mice has contributed to mitochondrial dysfunction of hippocampal neurons [84]. However, in AD animal models, inhibition of Drp1 could also improve learning and memory and prevent mitochondrial fragmentation, lipid peroxidation, and Aβ deposition in the brain [85]. All these contrasting observations suggest that the molecular event causing an imbalance in mitochondrial dynamics, especially in Drp1 regulation, is very complex and poorly understood to be attributed to AD progression. 

Another risk factor for AD progression is dysregulation of mitophagy. Results from animal, cellular models, and patients with AD suggest that impaired mitophagy contributes to synaptic dysfunction and cognitive deficits by triggering Aβ and Tau accumulation, thus promoting oxidative damage and cellular energy deficits [86]. As compared to normal subjects, the mitophagy levels were reduced by 30–50% in hippocampal brain samples of AD patients [87]. Interestingly, retrograde transport of distal damaged mitochondria to the neuronal soma is also impaired in AD patients [88]. Wang et al. reported that DISC1, a main protein regulating axonal mitochondria trafficking, is less abundant in AD patients [89]. Interestingly, a reduction of the autophagic ATG5 factor and of Parkin levels in the serum [90] was reported in the peripheral fluids of AD patients. Moreover, a decrease of Parkin concomitant with an increase of PINK1 and LC3 mRNA levels was observed in AD patients’ peripheral blood [91]. The study from Wang et al. delineates that overexpression of parkin ameliorates impaired mitophagy and promotes the removal of damaged mitochondria in Aβ-treated cells, as demonstrated by increased membrane potential (Δϕm), ETC complex activity, and ATP level [92]. The current studies have found that mice treated with Urolithin A, a mitophagy activator, are able to better remember and improve learning and reduce memory deterioration, remove Aβ from neurons, and prevent the onset of cognitive deficits associated with pathological Aβ deposition in the 3 × Tg-AD mouse models [93,94].

Biochemical studies have indicated that mitochondrial DNA defects are associated with depressed activities of all ETC complexes, especially with a dramatic reduction in cytochrome c oxidase (COX) activity, in AD brains [95]. Mitochondrial deficits have been found in both the neurons and astrocytes of AD brains [96,97], indicating that both neurons and astrocytes may be damaged by ROS. Two free radicals, H_2_O_2_ and O_2_^•−^, from the mitochondrial respiratory chain may be released into the cytoplasm and cause the oxidation of cytoplasmic molecules. In addition to COX, the other two key mitochondrial enzymes of oxidative metabolism, α-ketoglutarate dehydrogenase complex and pyruvate dehydrogenase complex, are also deficient in the AD brain [98]. Hirai et al. used postmortem brain specimens as a research model and found increased oxidative damage in AD patients, with a significant increase in mtDNA in the pyramidal neurons and cytochrome oxidase in the neuronal cytoplasm [97]. Such an increase in normal mitochondria and degraded mitochondrial cell bodies may cause enhanced oxidative damage. The study of Hirai et al. indicated that such mitochondrial degradation may play a vital role in AD progression.

Mitochondrial trafficking is also impaired in AD because synaptic mitochondria need to be synthesized in the cell bodies of neurons [99]. As described above, if the mitochondria of the cell body are damaged or degraded, these dysfunctional mitochondria are then transferred to synaptic terminals via natural trafficking. Thus, those defective mitochondria may not produce enough ATP. Evidence has demonstrated that Aβ may be the main cause of synaptic mitochondrial dysfunction in AD [100]. Mungarro-Manchaca et al. observed that the Aβ peptide induces ultrastructural changes (mitochondrial swelling and intense small synaptic vesicle depletion) and potentiates mitochondrial dysfunction upon ryanodine treatment [101]. These changes were concomitant with a reduced content of synaptophysin and actin proteins [101]. From the proteomic data, Gillardon et al. observed that Aβ oligomers are detected in synaptic mitochondrial fractions in AD transgenic Tg2576 mice, where the energy metabolism is also decreased [102]. They also found that mitochondrial alterations occur before amyloid plaque deposition, indicating that mitochondria are early targets of Aβ aggregates [102]. These findings suggest that the Aβ peptide may contribute to synaptic mitochondrial damage in AD, leading to low ATP production and, finally, synapse degeneration. Synapses are the sites of high ATP demand and are essential for exocytosis of neurotransmitters. In addition, synaptic terminals are also important for the release of Ca^2+^, especially in post-tetanic potentiation [103]. Therefore, mitochondrial trafficking to synaptic terminals is necessary to maintain neural functions. However, synaptic mitochondria are “older” than cell body mitochondria due to neutral trafficking, indicating that synaptic mitochondria may have more damage than cell body mitochondria [104]. Ultimately, the increased oxidative damage may affect neurotransmission and contribute to synaptic damage and loss, which are responsible for cognitive decline in AD progression.

The clinical symptoms of AD include the accumulation of plaques and tangles, leading to the damage and destruction of synapses and causing loss of memory and cognition. However, the foundational soluble blocks of these structures are Aβ peptides and tau proteins for plaques and tangles, respectively. In AD patients or animal models, Aβ accumulation (both the 1–40 and 1–42 forms) is significantly increased in the mitochondrial matrix [105]. In a culture system, the addition of Aβ to primary cortical neurons could induce ATP depletion, impair mitochondrial respiration, and increase ROS production [106,107]. Furthermore, Aβ exposure could reduce complex IV activity in an isolated mitochondria system [108] and increase cell death by inducing the formation of permeability transition pores [109]. Previous studies have demonstrated that the two mitochondrial proteins, amyloid binding alcohol dehydrogenase (ABAD) and Cyclophilin D (CypD), are able to mediate the accumulation and toxicity of Aβ peptides [110,111]. Furthermore, Aβ peptides may interact with α-synuclein as oligomers and accumulate in the mitochondrial and plasma membranes to activate the apoptosis cascade through the release of cytochrome *c* [112,113]. Jara et al. observed that tau deletion in hippocampal cells may decrease cellular oxidative damage, favor a mitochondrial profusion state, and inhibit mitochondrial permeability transition pore formation [114]. Furthermore, the current study from Yokoyama et al. has provided a comprehensive overview of the mouse models of AD [115], where we summarized the mitochondrial dysfunction or damage from those AD mouse models in Table 1. In the early stages of degeneration, the dopamine neurons in the Tg2576 mice demonstrated accumulated mitochondria damage due to free Ca^2+^ increase [116]. Reduced Drp1 in Tg2576 mice decreases soluble Aβ production in AD progression by reducing mitochondrial dysfunction, maintaining mitochondrial dynamics, and enhancing mitochondrial biogenesis and synaptic activity [117]. In J20 mice, Transglutaminase type 2 can interact with Aβ to form toxic aggregation then induce an acute increase in intracellular Ca^2+^, and an increased mitochondrial Ca^2+^ overload, thus affecting ER-mitochondria cross talk [118,119]. Increased basal and coupled respiration in the hippocampus along with a decreased Complex II-dependent respiratory activity were observed in TgCRND8 female mice [120]. APP/PS1 transgenic mice, the AD animal model we described in the previous section, showed increased cognitive impairment, hippocampal neuron mitochondrial damage, and autophagosome accumulation with aging [121]. The 5 × FAD mouse models had a significant decrease in ATP levels in the hippocampus [122] and decreased complex I, III, and IV with increased complex V relative to wild-type mice by mitochondrial proteomic analysis [123]. Increased carbonyls and dysregulated activity and content of mitochondrial enzymes, such as citrate synthase, MnSOD, and cytochrome *c* oxidase, were observed in PS19 mice [124]. Mitochondrial distribution is disrupted in somata and neurites with age in rTg4510 mice [125]. 3 × Tg AD mice are characterized by impaired bioenergetic function, decreased Ca^2+^ buffering capacity, defects in the oxidative phosphorylation system, and dysfunction of mitochondrial respiratory function in brain cortical mitochondria [126,127]. Interestingly, the mitochondrial function can be improved in the hippocampus of tau^−/−^ mice by providing more energy to the synapses [128]. Taken together, research on human AD patients and in vitro animal models has shown that all the key functions of mitochondria are influenced. The mitochondrial spiral, including impaired brain energy metabolism, ROS production, and disturbed Ca^2+^ homeostasis, indicates a role in the development of neurodegenerative diseases, particularly AD [128]. Due to maternal transmission, mitochondria are also critical in AD pathogenesis [129]. New evidence also specifically indicates a rationale for adopting mitochondrial maintenance as a target for AD therapy. 

## 5. The Therapeutic Strategies for AD by Maintaining Mitochondria Function

AD is a complex disease, and its treatment requires multiple approaches targeting the formation/clearance of Aβ and hyper-phosphorylated tau, supporting and stabilizing the remaining neuronal networks, and protecting the potentially sensitive mitochondria. However, given the wealth of evidence of mitochondrial dysfunction in aging and neurodegenerative diseases, a reasonable treatment strategy is to boost mitochondrial function for patients with AD. Such therapeutic strategies should include the aims of developing molecules for targeting ROS production and boosting ATP levels in mitochondria, which will ultimately increase the synaptic branching of neurons, synaptic outgrowth, and neuronal connectivity. Treatment or diet supplementation with antioxidants seems to be suitable for patients with AD. To date, there are several antioxidant drugs that have been considered in human trials to treat or prevent AD, but it needs to be noted that no therapy has been shown to arrest or even change the slope of decline in disease progression. The best well-known antioxidant in trial for AD is vitamin E (α-tocopherol). In 1997, a double-blind, placebo-controlled, randomized, multicenter trial of selegiline and vitamin E was published by the Alzheimer’s Disease Cooperative Study (ADCS) [130]. A total of 341 patients with AD of moderate severity were randomly assigned to placebo, selegiline, vitamin E, or both of the latter for an average follow-up of 2 years. However, no benefit of either selegiline or vitamin E on AD progression was seen without normalization for this baseline difference. Beneficial delays in disease progression on account of vitamin E or selegiline were only observed upon adjusting for baseline cognition, casting doubt on the statistical adjustment. Later, a total of 769 subjects with mild cognitive impairment randomly received vitamin E, selegiline, or placebo and were followed for 3 years. Although selegiline reduced AD risk for 1 year in all the patients and for all 3 years in subjects with the APOE4 allele and a slowing of hippocampal atrophy [130], vitamin E had no impact on the rate of conversion and progression to AD at any time point [131]. 

The kinetic problem of vitamin E may yet be observed to cause no effect on AD. Effective antioxidants should react with oxidants faster than oxidants react with endogenous molecules. Furthermore, although levels of CSF are increased upon vitamin E supplementation, it is not known whether the brain levels significantly increase [132]. Recent studies of AD mouse models and antioxidant supplements showed that vitamin E decreased Aβ pathology and ameliorated cognitive deficits [133,134]. However, the results of antioxidant supplements on elderly persons and AD patients were mixed, and most studies did not find the hoped-for benefits [135,136]. Some county-level studies showed no significant reduction in the risk of AD development treated with natural antioxidants, such as vitamins E and C [137,138,139]. Other studies found a reductive risk effect of antioxidant supplements on AD patients [132,140]. One reason for these mixed findings may be that such natural antioxidants might not cross the BBB, and therefore, they cannot reach the mitochondria to neutralize oxidative stress. In the last decade, some antioxidants have been developed to increase their delivery into mitochondria, including the triphenylphosphonium-based antioxidants (MitoQ, MitoVitE, and MitoPBN) [141]. Studies of ALS transgenic mice found that these mitochondria-targeted antioxidants enter the mitochondria at a several-hundred-fold rate and decrease mitochondrial toxicity [90,142]. MitoQ can improve cognition, prevent synaptic loss, and reduce oxidative stress in AD transgenic mouse models [143,144]. In vitro, MitoVitE could attenuate paclitaxel-induced mitochondrial damage and play as an antioxidant in rat dorsal root ganglion cells [145]. Moreover, MitoPBN treatment promotes mitochondrial biogenesis and increases mitochondria number through the PGC-1α pathway in liver cells, indicating the efficiency in mitochondrial functions [146]. Those mitochondria-targeted antioxidants are not all used in clinical trial for AD patients, instead of MitoQ (https://clinicaltrials.gov/study/NCT03514875 (accessed on 27 September 2023)). Therefore, those in vivo and in vitro studies demonstrate that those antioxidants may be potential drug candidates for treating AD patients.

**Figure 3:**
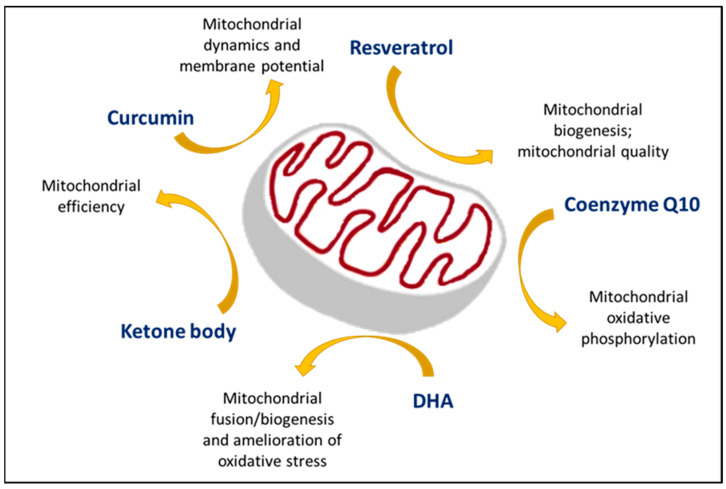
The different effects of nutraceutical supplements on mitochondria. The combination of mitochondrial nutrients is necessary in AD treatment approaches.

Moreover, the maintenance of mitochondrial function is another therapeutic approach for AD. Supplementation with nutraceuticals (curcumin, resveratrol, Coenzyme Q10, docosahexaenoic acid (DHA)) or ketone body treatment could boost mitochondrial functions [147]. Curcumin enhanced neuroplasticity and improved mitochondrial dynamics and membrane potential [148]. The administration of CoQ10 could improve mitochondrial function by altering the flow of calcium [149]. Resveratrol could upregulate SIRT1, ERK, and AMPK (PGC-1α) signaling to further contribute to mitochondrial functions and improve neuronal renewal [150]. DHA plays an antioxidant role and increases mitochondrial fusion, inhibits mitophagy, or promotes biogenesis due to an increase of p62, OPTN, PINK1, and PGC-1α [151]. The effects of nutraceutical supplementation on mitochondria are summarized in Figure 3 and Table 2.

## 6. Conclusions

There is now plenty of evidence suggesting that aging and age-related neurological diseases are highly associated with mitochondria dysfunction. Mitochondrial and synaptic dysfunctions represent the early pathological features of AD in the brain, indicating that maintaining mitochondrial function could be a potential therapeutic strategy for the early stages of AD. Mitochondrial dysfunction may lead to multiple adverse outcomes, including increased oxidative stress, decreased ATP production, and enhanced abnormal protein–protein interactions. Since the integrity and function of mitochondria represent the main factors for AD treatment, future treatment approaches should involve a combination of mitochondrial nutrients in both the prevention and treatment of AD.

## Figures and Tables

**Table 1:** Effects of Mitochondrial Function during Disease Progression in Various Representative AD Model Mice.

Model Mouse	Gene Mutation	Effect on Mitochondrial Functions	References
Tg2576	*APP* (KM670/671NL)	The dopamine neurons in the Tg2576 accumulate damaged mitochondria at the onset of degeneration	[116,117]
J20	*APP* (KM670/671NL, V717F)	Increased mitochondrial Ca^2+^ overload Early synaptosomal mitochondrial dysfunction	[118,119]
TgCRND8	*APP* (KM670/671NL, V717F)	Increased basal and coupled respiration in the hippocampus along with a decreased Complex II-dependent respiratory activity	[120]
APP/PS1	*APP* (KM670/671NL) *PSEN1* (delta9)	Increased hippocampal neuron mitochondrial damage	[121]
5×FAD	*APP* (KM670/671NL, V717I, I716V) *PSEN1* (M146L, L286V)	Decreased energy metabolism and mitochondrial biogenesis defects.	[122,123]
PS19	*MAPT* 1N4R tau (P301S)	Increased carbonyls and dysregulated the activity and content of mitochondrial enzymes	[124]
rTg4510	*MAPT* 0N4R tau (P301L)	Mitochondrial distribution is disrupted	[125]
3×Tg	*APP* (KM670/671NL) *MAPT* 0N4R tau (P301L) *Psen1* (M146V knock-in)	Impaired bioenergetic function, decreased Ca^2+^ buffering capacity, defects in oxidative phosphorylation system, and dysfunction of mitochondrial respiratory function	[126,127]

**Table 2:** The Summarized Nutraceutical Supplementation for Mitochondria Includes Structures, Natural Sources, and Mitochondrial Benefits.

Nutraceutical Supplements	Structure	Source	Mitochondria Benefits	References
Ketone body	Acetoacetate (AcAc) and 3-beta-hydroxybutyrate (major)	Oxidation of nonesterified or free fatty acids (FFAs) by the liver	Mitochondrial efficiency	[147]
Curcumin	1,7-bis(4-hydroxy-3-methoxyphenyl)-1,6-heptadiene-3,5-dione	Rhizome of Curcuma longa (turmeric) and in others Curcuma spp	Mitochondrial dynamics and membrane potential	[148]
Coenzyme Q10	1,4-benzoquinone	Oily fish (such as salmon and tuna), organ meats (such as liver), and whole grains	Mitochondrial oxidative phosphorylation	[149]
Resveratrol	3, 5, 4′-trihydroxystilbene	Grapes, apples, blueberries, plums, and peanut	Mitochondrial biogenesis; mitochondrial quality	[150]
DHA	Docosahexaenoic acid	Cold-water, fatty fish and seaweed	Mitochondrial fusion/biogenesis and amelioration of oxidative stress	[151]
